# Comparison of Homologous and Heterologous Booster SARS-CoV-2 Vaccination in Autoimmune Rheumatic and Musculoskeletal Patients

**DOI:** 10.3390/ijms231911411

**Published:** 2022-09-27

**Authors:** Dániel Honfi, Nikolett Gémes, Enikő Szabó, Patrícia Neuperger, József Á. Balog, Lajos I. Nagy, Gergely Toldi, László G. Puskás, Gábor J. Szebeni, Attila Balog

**Affiliations:** 1 Department of Rheumatology and Immunology, Faculty of Medicine, Albert Szent-Gyorgyi Health Centre, University of Szeged, H6725 Szeged, Hungary; 2 Biological Research Centre, H6726 Szeged, Hungary; 3 PhD School in Biology, University of Szeged, H6726 Szeged, Hungary; 4 Avidin Ltd., H6726 Szeged, Hungary; 5 Liggins Institute, University of Auckland, Auckland 1023, New Zealand; 6 Department of Physiology, Anatomy and Neuroscience, Faculty of Science and Informatics, University of Szeged, H6726 Szeged, Hungary; 7 CS-Smartlab Devices, H7761 Kozármisleny, Hungary

**Keywords:** SARS-CoV-2 booster vaccination, rheumatic and musculoskeletal diseases, anti-Spike (RBD) antibodies, SARS-CoV-2 specific T-cell immunity, SARS-CoV-2 specific peripheral B-cell memory

## Abstract

Vaccination against SARS-CoV-2 to prevent COVID-19 is highly recommended for immunocompromised patients with autoimmune rheumatic and musculoskeletal diseases (aiRMDs). Little is known about the effect of booster vaccination or infection followed by previously completed two-dose vaccination in aiRMDs. We determined neutralizing anti-SARS-CoV-2 antibody levels and applied flow cytometric immunophenotyping to quantify the SARS-CoV-2 reactive B- and T-cell mediated immunity in aiRMDs receiving homologous or heterologous boosters or acquired infection following vaccination. Patients receiving a heterologous booster had a higher proportion of IgM+ SARS-CoV-2 S+ CD19+CD27+ peripheral memory B-cells in comparison to those who acquired infection. Biologic therapy decreased the number of S+CD19+; S+CD19+CD27+IgG+; and S+CD19+CD27+IgM+ B-cells. The response rate to a booster event in cellular immunity was the highest in the S-, M-, and N-reactive CD4+CD40L+ T-cell subset. Patients with a disease duration of more than 10 years had higher proportions of CD8+TNF-α+ and CD8+IFN-γ+ T-cells in comparison to patients who were diagnosed less than 10 years ago. We detected neutralizing antibodies, S+ reactive peripheral memory B-cells, and five S-, M-, and N-reactive T-cells subsets in our patient cohort showing the importance of booster events. Biologic therapy and <10 years disease duration may confound anti-SARS-CoV-2 specific immunity in aiRMDs.

## 1. Introduction

Vaccination supporting both humoral and cellular immunity has emerged as an important strategy to relieve the rates and severe outcomes of Coronavirus Disease-19 (COVID-19) infection [1]. Various vaccines approved in different countries have been shown to be efficacious in reducing infection rates, disease severity, and mortality against Severe Acute Respiratory Syndrome Coronavirus-2 (SARS-CoV-2) in the whole human population [2,3,4]. Currently available data suggest that patients with aiRMDs have a moderately higher prevalence of SARS-CoV-2 infections, and a higher risk of hospitalization and mortality from COVID-19 compared to the general population [5,6,7]. Nevertheless, vaccines for SARS-CoV-2 are effective in patients with aiRMDs without presenting significant safety issues or leading to disease flares [8,9,10]. Furthermore, two doses of vaccines lead to significantly better outcomes of breakthrough COVID-19 compared with unvaccinated patients [11]. Data also supported that underlying aiRMDs even in patients in remission or with associated use of immune modifying drugs could attenuate responses to SARS-CoV-2 vaccination [12,13].

In the second half of 2021, several studies documented the waning effectiveness of vaccines over time, especially against the new, more infectious Delta variant and to less extent against COVID-19-related death [14,15]. To maintain protection against emerging new waves and variants, booster vaccine doses were given in several countries in the summer of 2021 [14,16]. The safety and benefit of booster vaccination have been demonstrated by a number of studies including the general population [17,18] and RMD patients [8,19]. However, data concerning the humoral and cellular immune response of booster homologous and heterologous vaccination in patients with aiRMDs are limited. The complex immunogenicity of patients with aiRMDs who got two doses of vaccines and were later infected by SARS-CoV-2 has only been partially investigated.

In spring 2021, we had the opportunity to vaccinate our RMD patient’s frontline with two doses of one type of COVID-19 vaccine in our center selected from 5 available vaccines (BBIBP-CorV; Gam-COVID-Vac; AZD1222; BNT162b2; mRNA-1273) [12]. In autumn 2021, patients received booster vaccination with BNT162b2 vaccine 6 months after the second dose or underwent SARS-CoV-2 infection as a third exposure to SARS-CoV-2 antigens, with significantly different immunogenicity reported in our current study. We aimed to compare humoral and cellular immune responses after either homologous or heterologous vaccination among patients with aiRMDs at their third vaccination with BNT162b2 or with two vaccinations followed by COVID-19 infection. Here, we determined the neutralizing anti-SARS-CoV-2 antibody levels and applied flow cytometric immunophenotyping for quantifying the SARS-CoV-2 reactive B-, or T-cell mediated immunity in aiRMDs receiving homologous (Hom.), heterologous (Het.) vaccines or became infected (Inf.) as a booster event.

## 2. Results

### 2.1. Clinical Characteristics

There was no significant flare or relapse among patients with aiRMDs during the observation period following the third vaccination or infection. No alteration or adjustment of disease-modifying antirheumatic drugs (DMARDs) therapy or the dosage of glucocorticoids (GCs) following vaccination was necessary for any of the patients. None of the participants were diagnosed with COVID-19 infection from the patients’ group with booster vaccination. The infection was detected by Real-Time Quantitative Polymerase Chain Reaction (RT-qPCR) technique. All COVID-19-infected patients in the third group suffered from mild clinical symptoms without hospitalization. The safety profile of vaccines amongst patients is presented in Table 1. Only mild or moderate local and systemic side effects appeared in the patients following the first, second, and booster vaccination with a tendency of increasing occurrence following the third booster vaccination (Table 1).

### 2.2. Humoral and B-Cell Mediated Anti-SARS-CoV-2 Response

**Figure 1:**
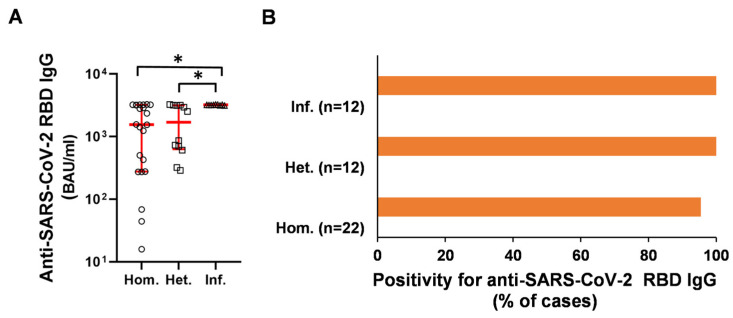
Neutralizing anti-SARS-CoV-2 RBD IgG antibody levels in the three patient groups. (**A**) Subjects receiving homologous (circles: Hom., *n* = 22), or heterologous (squares: Het., *n* = 12) booster vaccination or subjects infected after 2 doses of vaccines (triangles: Inf., *n* = 12) were assayed as described in the 4.4 Materials and Methods section. Horizontal lines—median, whiskers—interquartile range (Q1–Q3). (**A**) * *p* < 0.05 Hom. vs. Inf.; * *p* < 0.05 Het. vs. Inf. (**B**) The percentage of responders was 95.5% for Hom. and 100–100% for Het. and Inf. groups.

The virus neutralizing, anti-receptor binding domain (anti-RBD) specific IgG antibody levels were higher in patients who became infected with SARS-CoV-2 following primary complete vaccination protocol than in those who received a homologous and heterologous booster vaccine (3207 [3164–3235] vs. 1553 [276–3211] and 1689 [631–3162], (median [Q1–Q3]) BAU/mL, (* *p* < 0.05), respectively) (Figure 1A). Positive (>21.8 BAU/mL) neutralizing anti-RBD IgG antibody response was observed in 95.5%, 100%, and 100% of the homologous and heterologous as well as the SARS-CoV-2 infected groups (Figure 1B), respectively.

**Figure 2:**
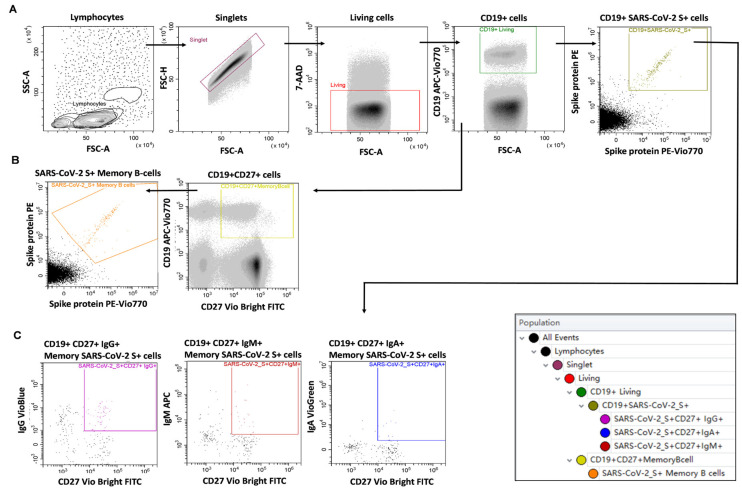
Gating strategy of the immunophenotyping to determine the SARS-CoV-2 S-protein reactive peripheral (**A**) CD19+ B-cells, (**B**) CD19+CD27+ memory B-cells, (**C**) the SARS-CoV-2 S-protein reactive peripheral IgG, IgM, or IgA positive CD19+CD27+ memory B-cells. After the withdrawal of peripheral blood, PBMCs were purified and labeled with a cocktail of antibodies and fluorescently labeled spike (S) protein as described in the 4.5 Materials and Methods section. Reactive cells were acquired and quantified on a flow cytometer. Population hierarchy corresponds to the gating hierarchy on the right. Cell debris and aggregates were excluded by manual gating determining single cells as singlets. To avoid the analysis of the unspecific labeling of dead cells, living cells were gated as negative for the 7-AAD viability dye. The gate of lymphocytes was determined by the CD19 positivity of living singlets. Living singlets of lymphocytes were analyzed further.

Next, we aimed to further investigate the humoral immune response, namely, the SARS-CoV-2 Spike (S) protein reactive peripheral B-cells and memory B-cells of the aiRMDs patients. The S protein reactive CD19+ B-cells and SARS-CoV-2 S protein reactive CD19+CD27+ peripheral memory B-cells were determined by flow cytometry (Figure 2).

**Figure 3:**
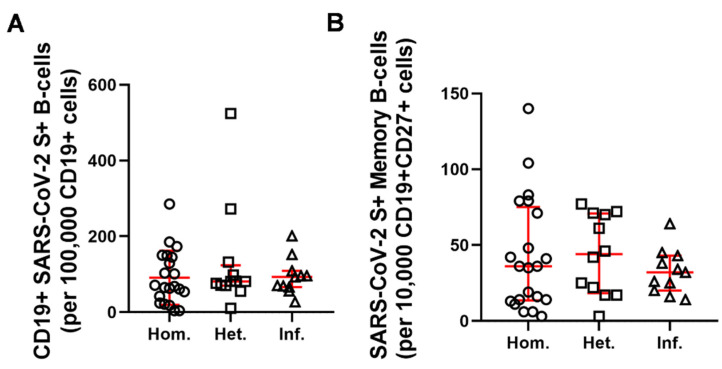
Quantitative analysis of peripheral SARS-CoV-2 S-protein reactive CD19+ B-cells (**A**) or CD19+CD27+ memory B-cells (**B**) of RMD patients. (circles: Hom. *n* = 21, squares: Het. *n* = 12, triangles: Inf. *n* = 11), Horizontal lines—median, whiskers—interquartile range.

Single-cell flow cytometric immunophenotyping identified S reactive CD19+ B-cells in relative numbers such as 68 [33–147], 81 [70–123], and 93 [66–109], (median [Q1–Q3]) in the Hom., Het., and Inf. groups among 1 × 10^5^ cells of the parental whole CD19+ peripheral B-cell population, respectively (Figure 3A). The number of S-specific peripheral CD19+CD27+ memory B-cells were 36 [13–75], 44 [18–70], and 31 [20–43], (median [Q1–Q3]) in the Hom., Het., and Inf. groups among 1 × 10^4^ cells of the parental whole CD19+CD27 peripheral memory B-cell population, respectively (Figure 3B). There was no statistically significant difference between booster vaccination types and booster infection in terms of the relative numbers of reactive B-cells. The percentage of responder cases with a positive memory B-cell response, namely with above 40 S-reactive CD19+CD27+ peripheral memory B-cells/1 × 10^4^ parental population were 42.9%, 58.3%, and 36.3% for the Hom., Het., or Inf. groups, respectively.

The IgM and isotype switched IgG, or IgA positive SARS-CoV-2 S protein reactive CD19+CD27+ peripheral memory B-cells were further determined by flow cytometry (Figure 2C).

**Figure 4:**
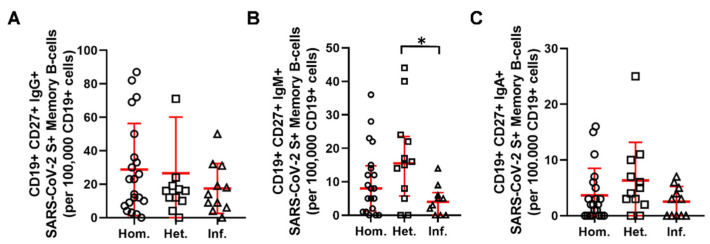
The proportion of (**A**) IgG+, (**B**) IgM+, (**C**) IgA+ SARS-CoV-2 S-antigen reactive memory (CD19+CD27+) B-cells in the three patient groups (circles: Hom. *n* = 21, squares: Het. *n* = 12, triangles: Inf. *n* = 11). Horizontal lines—median, whiskers—interquartile range. * *p* < 0.05 Het. vs. Inf.

Isotype switched IgG positive S reactive CD19+CD27+ memory B-cells were 23 [8–43], 16 [10–23], and 14 [6–31], (median [Q1–Q3]) among 1 × 10^5^ peripheral CD19+B-cells in the Hom., Het., and Inf. groups, respectively (Figure 4A). Patients receiving a heterologous booster had a significantly higher proportion of IgM+ SARS-CoV-2 S antigen memory (CD19+CD27+) B-cells in comparison to those who acquired SARS-CoV-2 infection (15.5 [6–23] vs. 4 [0–7] (* *p* < 0.05), (median [Q1–Q3]) cells in 1 × 10^5^ CD19+ cells) (Figure 4B). Isotype switched IgA positive S reactive CD19+CD27+ memory B-cells were 2 [0–5], 4 [2–9], and 3 [0–5], (median [Q1–Q3]) among 1 × 10^5^ peripheral CD19+B-cells in the Hom., Het., and Inf. groups, respectively (Figure 4C).

**Figure 5:**
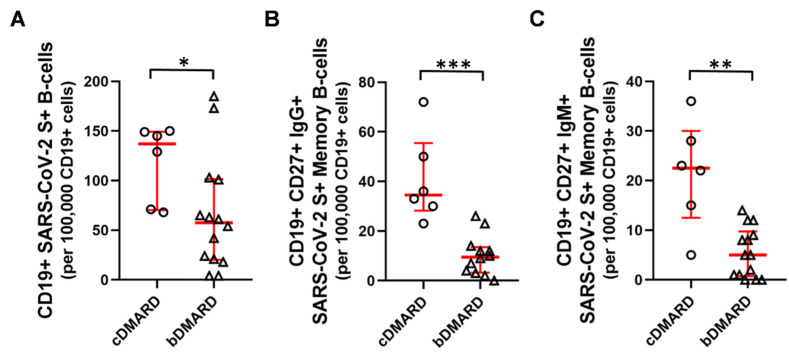
The proportion of CD19+ SARS-CoV-2 S+ B-cells (**A**), IgG+ (**B**), or IgM+ (**C**) SARS-CoV-2 S antigen reactive memory (CD19+CD27+) B-cells within patients receiving a homologous booster split according to their medication. Horizontal lines—median, whiskers—interquartile range. (circles: cDMARD *n* = 6, triangles: bDMARD *n* = 15). * *p* < 0.05; ** *p* < 0.01; *** *p* < 0.001.

Within the group receiving a homologous booster, subgroup analyses revealed the weakening effect of bDMARD therapeutic intervention on humoral immunity. The proportion of reactive CD19+ SARS-CoV-2 S+ B cells in the cDMARD group was 137 [70–149] vs. 57 [20–101], (median [Q1–Q3]) (* *p* < 0.05) per 1 × 10^5^ CD19+ B-cells in the bDMARD group (Figure 5A). As well as that of IgG+ CD19+CD27+ SARS-CoV-2 S+ memory B-cells in the cDMARD group were 35 [28–55] vs. 9 [3–13], (median [Q1–Q3]) (*** *p* < 0.001) per 1 × 10^5^ CD19+ B-cells in the bDMARD group (Figure 5B). The IgM+ SARS-CoV-2 S+ antigen memory (CD19+CD27+) B-cells were also higher in patients on cDMARD therapy, 23 [12–30] vs. 5 [1–10] (median [Q1–Q3]) (** *p* < 0.01) per 1 × 10^5^ CD19+ B-cells in comparison to those treated with bDMARDs (Figure 5C).

### 2.3. T-Cell Mediated Anti-SARS-CoV-2 Response

**Figure 6:**
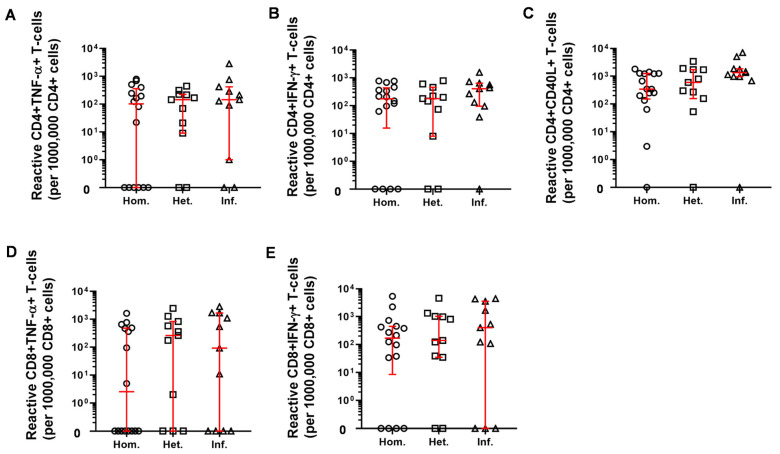
The quantitation of SARS-CoV-2-derived S-, M-, N-reactive peripheral T-cells ex vivo. After the withdrawal of peripheral blood, PBMCs were purified and plated for stimulation ex vivo for 16h with S-, M-, and N-peptide pools. After incubation cells were labeled with a cocktail of antibodies as described in the 4.6 Materials and Methods section. Reactive cells (**A**) CD4+/TNF-α+, (**B**) CD4+/IFN-γ+, (**C**) CD4+/CD40L+, (**D**) CD8+/TNF-α+, and (**E**) CD8+/IFN-γ+ were acquired and quantified on a flow cytometer. (circles: Hom. *n* = 16, squares: Het. *n* = 11, triangles: Inf. *n* = 11). Horizontal lines—median, whiskers—interquartile range.

Next, flow cytometric immunophenotyping was used for assaying cellular immunity, namely the SARS-CoV-2 reactive peripheral T-cell numbers. PBMCs were plated and stimulated ex vivo with SARS-CoV-2 derived S-, M- (Membrane protein), and N (Nucleocapsid protein) peptide pool. No differences were identified between the three patient groups with regards to the proportion of reactive CD4+TNF-α+, CD4+IFN-γ+, CD4+CD40L+, CD8+TNF-α+ or CD8+IFN-γ+ T-cell subsets (Figure 6).

**Figure 7:**
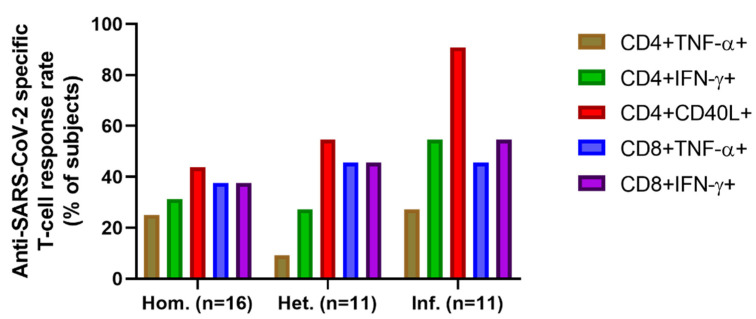
The rate of positive T cell responses within the investigated T cell subsets in the three patient groups.

The rate of positive T-cell responses within these cell subsets in the three patient groups is presented in Figure 7. The percentages of subjects with positive CD4+TNF-α responses were 25%, 9.1%, and 27.3% in the Hom., Het., and Inf. Groups, respectively. The frequency of responder cases of CD4+IFN-γ+ SARS-CoV-2 reactive T-cell response was higher than the TNF-α reaction at 31.3%, 27.3%, and 54.5% in the same order of groups. The response rate to booster event was the highest in the S-, M-, N- reactive CD4+CD40L+ T-cell parameter: 43.8% (Hom.), 54.5% (Het.), and the outstanding 90.9% were registered in the infection boosted group (Figure 7). The responders to the booster event were 38% (Hom.) 46–46% (Het. and Inf.) for CD8+TNF-α+ T-cell activation and 38% (Hom.), 46% (Het.), 55% (Inf.) for CD8+IFN-γ T-cell mediated SARS-CoV-2 protective immunity (Figure 7).

**Figure 8:**
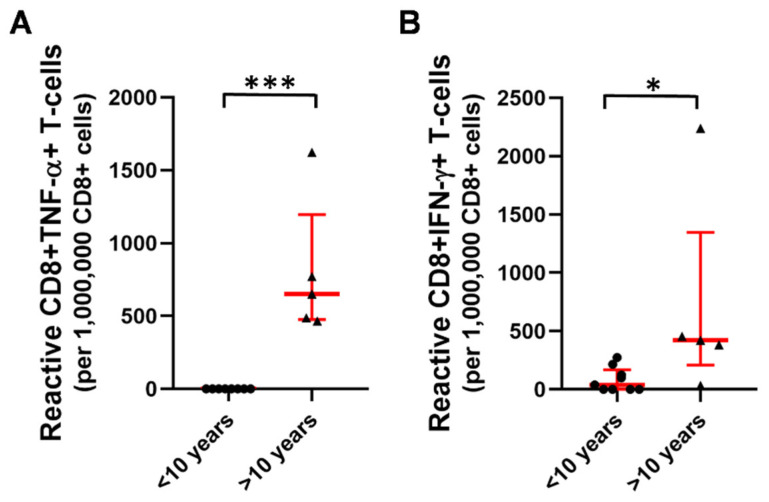
The proportion of (**A**) CD8+TNF-α+ and (**B**) CD8+IFN-γ+ T-cells within patients receiving a homologous booster split according to disease duration, (*n* = 9 for <10 years, *n* = 5 for >10 years), (bullets: <10 years, triangles: >10 years). Horizontal lines—median, whiskers—interquartile range. * *p* < 0.05; *** *p* < 0.001.

Patients with a disease duration of more than 10 years had higher proportions of CD8+TNF-α+ (0 vs. 651 [476–1197], *** *p* < 0.001) and CD8+IFN-γ+ T-cells (38 [0–170] vs. 422 [209–1347], * *p* < 0.05) in comparison to patients who were diagnosed less than 10 years ago within the homologous booster group (Figure 8). Similar, but statistically not significant trends were observed for the groups of patients receiving a heterologous booster (CD8+TNF-α+ (218 [0–940] vs. 360 [1–1513]; CD8+IFN-γ+ T-cells (474 [38–1101] vs. 552 [31–3680] for <10 years versus >10 years) or acquiring SARS-CoV-2 infection following primary vaccination (CD8+TNF-α+ (46 [0–208] vs. 1669 [6–2262]; CD8+IFN-γ+ T-cells (116 [0–195] vs. 1668 [266–4383] for <10 years versus >10 years).

## 3. Discussion

Here we report the results of one of the first single-center prospective studies conducted during the COVID-19 pandemic to investigate the complex humoral and cellular immune response followed by booster vaccination and infection of SARS-CoV-2 in aiRMDs patients. Our study also provides detailed information regarding the impact of aiRMDs, disease duration, and various immunosuppressive treatments on booster vaccine-induced or vaccination followed by infection-induced immunogenicity against SARS-CoV-2. We demonstrated that homologous and heterologous booster vaccination and infection as a booster event were immunogenic in patients with aiRMDs with variable B-cell and T-cell responses and an acceptable safety profile. These findings support the results of recent studies where considerable immunogenicity was induced by anti-SARS-CoV-2 booster vaccines [18,19].

In our previous work related to the current study, the rate of positive anti-RBD antibody response for patients with aiRMDs after 4 months of two dosages vaccination was only 55% for the inactivated viral vaccine BBIBP-CorV, 53% for the pooled data of adenovirus vector-based vaccines Gam-COVID-Vac and AZD1222, and 81% for the pooled data of mRNA vaccines BNT162b2 and mRNA-1273, respectively [12]. Our data reported here showed that following 4 months of the booster vaccination with the third dose of BNT162b2 or a ‘booster infection’, the positive (>21.8 BAU/mL) neutralizing anti-RBD IgG antibody response was outstanding in all three patient groups, 95.5%, 100% and 100% of the homologous and heterologous as well as the SARS-CoV-2 infected groups, respectively. Taken together booster vaccinations or SARS-CoV-2 infection after completing 2 doses of the vaccination can lead to the production of neutralizing antibodies still protective in RMD cases after 4 months of the third antigen exposition. Recent studies showed that booster vaccination further reduces the frequency of COVID-19-related hospital admissions and deaths in people with aiRMDs. Furthermore, the results suggest that booster COVID-19 vaccination has beneficial effects in patients with aiRMDs to a similar extent to what has been found for the general population [8].

Our data support the efficacy of booster vaccinations, independently from the type of vaccine used for the first two doses. Infection with mild symptoms can also be regarded as a booster event based on our results, which can be regarded as an alternative to booster vaccination. However, the IgG+ and IgA+ SARS-CoV-2 specific memory B-cell populations showed a statistically not significant but lower frequency in the Inf. group. The IgM+ SARS-CoV-2 S-antigen reactive memory (CD19+CD27+) B-cells showed significantly reduced frequency in the Inf. group. The authors may hypothesize that third exposure to SARS-CoV-2 antigens, as an infection may counteract the development of SARS-CoV-2 adaptive immune memory as it is reported via viral immune escape mechanisms such as the inhibition of type I and III IFNs [20,21].

Clinical activity of aiRMDs was not increased following booster vaccination, suggesting the safety of vaccination amongst these patients. Our previous results indicated the reduction of humoral immunity in patients receiving B-cell inhibitory therapy [12,13]. These conclusions were drawn based on antibody levels. We report reduced S-reactive peripheral memory B-cell numbers in patients receiving bDMARDs compared to those on cDMARDs. However, none of our patients received B-cell inhibitory treatment in our current study. Accordingly, following booster vaccination, the monitoring of humoral immune function is warranted not only in the case of B-cell inhibitory therapy, but also in the case of bDMARDs.

Pusnik et al. showed that SARS-CoV-2 infection-induced CD4+CD40L+ T-cells may support the development of S-reactive memory B-cells [22], in line with this we have shown the highest anti-RBD IgG production and the highest S-, M-, N-reactive CD4+CD40L+ T-cell frequency in the infection boosted group. Pusnik et al. also showed that COVID-19 recovered patients developed fewer but functionally superior spike-specific memory B cells which can explain that the S-reactive memory B-cells showed a lower frequency in the Inf. group in our study.

Several studies suggested dynamic changes in the T-cell phenotype of patients over time even in low disease activity or remission [23,24,25]. In our previous studies, the percentages of several circulating T-cell subtypes were prospectively measured in rheumatoid arthritis, ankylosing spondylitis, and inflammatory bowel diseases, respectively. The different T-cell repertoire was described in the three different investigated inflammatory diseases; however, the patient’s disease activity was low, or they were in remission. Furthermore, we found that different classes of targeted therapies have also different impacts on T-cell homeostasis although their efficacy and tolerability as assessed by clinical and routine laboratory values are identical [23,24,25]. Changes in T-cell phenotype seem to develop progressively during disease duration, even in inactive disease, and reflect ongoing effector T-cell differentiation and activation, along with the parallel compensatory increase in regulatory T-cells [24]. Patients with a disease duration of more than 10 years had higher proportions of SARS-CoV-2-specific CD8+TNF-α+ and CD8+IFN-γ+ T-cells in comparison to patients who were diagnosed less than 10 years ago. Authors speculate that in the earlier phase of aiRMDs, autoreactive T-cells might be present in a higher proportion, while in later phases of remission, the autoreactive CD8+ response might be less pronounced. Hence, upon infectious stimuli, virus-specific CD8+ T-cell responses may be increased.

Matula et al., recently showed that heterologous booster vaccination (BBIBP-CorV+BBIBP-CorV+BNT162b2) resulted in a comparable humoral and higher IFNγ-positive T-cell response than a homologous booster (BNT162b2+BNT162b2+BNT162b2) in the general population [26]. Our previous data harmonize with recent clinical and laboratory data concerning the necessity of a booster vaccination strategy [12,27]. Booster vaccination effectively increases the level of anti-SARS-CoV-2 antibodies without significant adverse events. Furthermore, patients who received a booster at least 5 months after the second dose of BNT162b2 had 90% lower mortality due to COVID-19 compared to those who did not receive a booster [28]. On the other hand, the peak titer of the anti-S protein neutralizing antibodies in individuals previously infected with SARS-CoV-2 and vaccinated with the BNT162b2 vaccine was more than 140 times higher than that before vaccination [29]. These data and our results suggested that the need for a booster might be delayed, and infection might be equivalent to the third booster dose in aiRMD patients who had already received two anti-SARS-CoV-2 vaccines.

In summary, our data draws attention to the importance of booster SARS-CoV-2 vaccines among aiRMDs patients. The third booster mRNA-based vaccine was similarly effective both in the homologous and heterologous groups compared to the infection-boosted patients. Overall, there was no hospitalization or death due to COVID-19 in the medical history of the studied aiRMDs.

## 4. Materials and Methods

### 4.1. Ethical Statement

The enrollment of patients was reviewed and approved by the Human Investigation Review Board of the University of Szeged under Project Identification Code 96/2021-SZTE-KREB. The patients provided their written informed consent to participate in this study. Subjects were informed about the study by a physician and acute SARS-CoV-2 infection was ruled out by RT-qPCR. Laboratory studies and interpretations were performed on coded samples lacking personal and diagnostic identifiers. The study adhered to the tenets of the most recent revision of the Declaration of Helsinki.

### 4.2. Study Population

The main characteristics of the study participants (46 patients with aiRMDs) are summarized in Table 2. All participants received two doses of the relevant vaccine in line with recommendations of the respective manufacturer of BBIBP-CorV (Sinopharm, Beijing Institute, Beijing, China); Gam-COVID-Vac (Sputnik V, Gamaleya Research Institute, Moscow, Russia); AZD1222 (ChAdOx1, University of Oxford and AstraZeneca, Cambridge, UK); BNT162b2 (Comirnaty, Pfizer-BioNtech, Mainz, Germany), then received booster vaccination with BNT162b2 vaccine 6 months after the second dose or underwent SARS-CoV-2 infection also 6 months after the second dose of vaccine. The homologous booster vaccinated group received three dosages of the BNT162b2 vaccine. Six out of 12, four out of 12, and two out of 12 patients received two doses of Gam-COVID-Vac, BBIBP-CorV, AZD1222 vaccine, and BNT162b2 as a third vaccine in the heterologous vaccinated group, respectively. Adult patients were enrolled during regular visits at the Department of Rheumatology and Immunology (University of Szeged, Hungary) based on the following inclusion criteria: rheumatoid arthritis (RA)—ACR/European League Against Rheumatism (EULAR) 2010 classification criteria [30]; psoriatic arthritis (PsA)—Classification Criteria for PsA [31]; axial spondyloarthritis (axSpA)—Assessment of SpondyloArthritis International Society classification criteria [32]; systemic lupus erythematosus (SLE)—ACR 1997 [33] or Systemic Lupus Erythematosus International Collaborating Clinics 2012 criteria [34]; systemic vasculitis, namely, large vessel vasculitis (LVV), antineutrophil cytoplasmic antibody-associated vasculitis (AAV), granulomatosis with polyangiitis (GPA) and eosinophilic GPA—Chapel Hill Consensus Conference definitions [35]; Sjögren syndrome (SS) ACR/EULAR 2016 criteria [36]; Behcet’s disease—International Criteria for Behcet’s Disease (ICBD) [37]. All patients were either in remission or had a low disease activity. All patients were on stable medication for at least the last eight weeks before enrolment. The medication and characteristics of the patients are summarized in Table 2. A stable dose of glucocorticoid (GC) ≤ 4 mg per day was permitted. All patients were instructed to discontinue all medications during the vaccination period for two weeks in the vaccinated groups and the infected group. Exclusion criteria for all groups were pregnancy and history of past vaccination allergy.

Table 2 Data are presented as mean ± SD or *n* (%). AAV, antineutrophil cytoplasmic antibody (ANCA)-associated vasculitis; aiRMD, autoimmune and inflammatory rheumatic and musculoskeletal disease; AxSpA, axial spondyloarthritis; LVV, large vessel vasculitis; PsA, psoriatic arthritis; RA, rheumatoid arthritis; SLE, systemic lupus erythematosus, SS, Sjögren syndrome; GC, glucocorticoids; cDMARD, conventional DMARD including methotrexate, leflunomide, azathioprine, chloroquine, cyclosporine, hidroxychloroquine; bDMARD, biological DMARD including tumor necrosis factor alpha inhibitors (10 patients with RA and 1 patient with AxSpa and 1 patient with PsA), interleukin 17 inhibitors (2 patients with PsA and 2 patients with AxSpa), interleukin 6 inhibitors (4 patients with RA); JAKi, janus kinase inhibitor.

### 4.3. Study Design

This prospective observational study was conducted at the Department of Rheumatology and Immunology of Albert Szent-Györgyi Medical School, University of Szeged, Hungary between October 2021 and March 2022. Adult patients with aiRMDs were recruited who received booster vaccination 6 months after the second dose or underwent SARS-CoV-2 infection 6 months after the second vaccine dose, all third events (exposition to SARS-CoV-2 antigens as booster vaccination or infection) occurred between October 2021 and November 2021. Peripheral blood and sera sampling was conducted after 4 months of the booster event between February–March 2022. 

The primary endpoint was the humoral and cellular immunogenicity of homologous booster vaccination: three doses of BNT162b2, heterologous booster vaccination: two doses of AZD1222, two doses of Gam-COVID-Vac, or two doses of BBIBPCorV plus a third dose of BNT162b2. The third group was SARS-CoV-2 infected after completing two doses of different homologous vaccination with BBIBPCorV, AZD1222, or BNT162b2, respectively. 

Secondary endpoints included: Effect of immunosuppressive treatment and patients’ clinical characteristics on the production of anti-RBD neutralizing antibodies and SARS-CoV-2 specific cellular immune response in aiRMD patients.

### 4.4. Measurement of SARS-CoV-2 Specific Antibodies

Measurement of SARS-CoV-2 anti-Spike (S) IgG antibodies was performed as described in detail previously [12]. Briefly, quantitative measurement of neutralizing anti-RDB specific IgG-type antibody titers was performed with the Siemens Advia Centaur XPT system using the Siemens Healthineers SARS-CoV-2 IgG assay (sCOVG) (Siemens Healthineers, Munich, Germany). Irsara et al., showed a proper correlation (r = 0.84) of the positive sCOVG assay results with virus neutralization capacity [38]. Measured Index Values were converted into WHO 20/136 approved international units of 1000 Binding Antibody Unit per milliliter (BAU/mL) using the following equation: (sCOVG Index) × 21.8 = BAU/mL, where the diagnostic cut-off value was 21.8 BAU/mL), assay sensitivity was 10.9 BAU/mL [12,39].

### 4.5. Measurement of SARS-CoV-2 Specific B-Cell Memory

Measurement of SARS-CoV-2 specific peripheral B-cell memory was performed according to the instruction of the manufacturer using the SARS-CoV-2 Spike B Cell Analysis Kit (Miltenyi Biotec, Bergisch Gladbach, Germany). Peripheral venous blood (8 mL) was taken into Lithium Heparin treated tubes (BD Vacutainer, Becton Dickinson). Peripheral blood mononuclear cells (PBMCs) were isolated by Ficoll density gradient centrifugation using Leucosep tubes (Greiner Bio-One, Kremsmünster, Austria). Cells were pelleted by centrifugation at 800× *g* for 20 min. The ring of PBMCs was harvested by pipetting and diluted with 15 mL PBS, then centrifuged at 350 g for 5 min. The supernatant (S/N) was removed. If necessary, red blood cells were lysed by 2 mL ACK solution (prepared in our laboratory: 0.15 M NaH_4_Cl, 10 mM KHCO_3_, 0.1 mM Na_2_EDTA, pH 7.4, Merck, Darmstadt, Germany) at room temperature (RT) for 2 min. Cells were washed with 15 mL PBS and centrifuged at 350 g for 5 min. The SARS-CoV-2 spike tetramer was prepared by incubating the Spike (S)-Biotin with Streptavidin PE or the Spike (S)-Biotin with Streptavidin PE—Vio 770, respectively at RT for 15 min. Cells were resuspended in 1 mL PBS and centrifuged at 350 g for 5 min. The pellet was resuspended in PEB buffer containing the antibody cocktail: anti-CD19 APC-Vio 770 (clone LT19), anti-CD27 VioBright FITC (clone M-T271), anti-IgG VioBlue (clone IS11-3B2.2.3), anti-IgA VioGreen (clone IS11-8E10), anti-IgM APC (clone PJ2-22H3) labeled S-proteins (S-Biotin Streptavidin-PE+S-Biotin Streptavidin-PE Vio 770, 1:2), and 7-AAD (2.6 µg/mL) in 100 µL total volume. Cells were incubated at 4°C for 30 min. Cells were washed in 1 mL PBS and centrifuged at 350 g for 5 min. Cells were resuspended in 550 µL PEB and a minimum of 1 × 10^6^ events were acquired in the lymphocyte gate on CytoFLEX S fluorescence-activated cell sorter (FACS) (Beckman Coulter, Brea, CA, USA). Manual gating was used to determine (1) CD19+ living singlets binding labeled S-proteins (CD19+ SARS-CoV-2 S+ cells), (2) CD19+CD27+ memory B-cells binding labeled S-proteins (SARS-CoV-2 S+ Memory B-cells), (3) CD19+ SARS-CoV-2 S+ cells that are IgG+CD27+ (CD19+ CD27+ IgG+ Memory SARS-CoV-2 S+ cells), (4) CD19+ SARS-CoV-2 S+ cells that are IgM+CD27+ (CD19+ CD27+ IgM+ Memory SARS-CoV-2 S+ cells), (5) CD19+ SARS-CoV-2 S+ cells that are IgA+CD27+ (CD19+ CD27+ IgA+ Memory SARS-CoV-2 S+ cells). Manual gating was performed in CytExpert (Beckman Coulter). Cell numbers in the reporting gates were normalized to parental populations: (1) the CD19+ SARS-CoV-2 S+ cells to 1 × 10^5^ CD19+ living singlets, (2) the SARS-CoV-2 S+ Memory B-cells to 1 × 10^4^ CD19+CD27+ living singlets, (3–5) the isotype-specific CD19+CD27+ Memory SARS-CoV-2 S+ B-cells were normalized to 1 × 10^5^ CD19+ living singlets. The cut-off value was 40 reactive (2) SARS-CoV-2 S+ Memory B-cells of 1 × 10^4^ parental CD19+CD27+ population.

### 4.6. Measurement of SARS-CoV-2 Specific T-Cell Mediated Immunity

Measurement of SARS-CoV-2 specific T-cell mediated immunity was performed as described in detail previously [12]. Briefly, measurement of SARS-CoV-2 Specific T-Cell Memory was performed according to the instruction of the manufacturer using the SARS-CoV-2 Prot_S T Cell Analysis Kit (PBMC) (Miltenyi Biotec). After the isolation of PBMCs and in vitro stimulation, staining with the antibodies, a minimum of 2 × 10^5^ CD3+ cells were acquired on CytoFLEX S FACS (Beckman Coulter). Manual gating was used to determine CD4+ or CD8+ T-cells within live CD14− CD20− CD3+ lymphocytes in CytExpert (Beckman Coulter). Reactive cells were gated as CD4+TNF-α+, CD4+IFNγ+, CD4+CD40L+, CD8+TNF-α+ and CD8+IFNγ+ upon S-, M-, N-stimuli. Cell numbers in the reporting gates were normalized to parental CD4+ or CD8+ cells (reactive cell number/parental cell number × 10^6^), then the background was normalized via subtraction of untreated from the stimulated. Finally, reactive cell numbers are shown in relation to 10^6^ CD4+ or CD8+ T-cells, the cut-off value was 400 reactive cells of 10^6^ parental population.

### 4.7. Statistics

Data are expressed as median [interquartile range, Q1–Q3]. Comparisons were made using Kruskal-Wallis and Mann-Whitney tests, as data were non-normally distributed according to the Kolmogorov-Smirnov test. Outliers were identified by the unsupervised ROUT method embedded in GraphPad Prism and were excluded from the analysis. *p* values less than 0.05 were considered significant. Statistics were calculated using the GraphPad Prism 8 software (GraphPad, San Diego, CA, USA).

## 5. Conclusions

Based on our current results and literature data [5], the evaluation of both B-memory cells and different T-cell phenotypes provides further information on the effects of certain immunosuppressive drugs and the clinical characteristics of patients with aiRMDs on the immunological reaction to vaccination. Patients receiving a heterologous booster had a higher proportion of IgM+ SARS-CoV-2 S+ CD19+CD27+ peripheral memory B-cells in comparison to those who acquired SARS-CoV-2 infection. Biologic therapy decreased the number of S+CD19+; S+CD19+CD27+IgG+; and S+CD19+CD27+IgM+ B-cells. The response rate to booster events in cellular immunity was the highest in the S-, M-, and N-reactive CD4+CD40L+ T-cell parameters: 43.8% (Hom.), 54.5% (Het.), and the outstanding 90.9% in the infection boosted group. Patients with a disease duration of more than 10 years had higher proportions of CD8+TNF-α+ and CD8+IFN-γ+ T-cells in comparison to patients who were diagnosed less than 10 years ago. Our study emphasizes that patients with aiRMDs undergoing the booster vaccination or infection following two doses of different COVID-19 vaccines have outstanding SARS-CoV-2 antibody production after the booster events.

## Figures and Tables

**Table 1:** Adverse events (AE) in aiRMD patients after the first, second, and third booster doses of the investigated vaccines. The number of mild or moderate local and systemic AE in aiRMDs was 14 (BNT162b2: *n* = 12, AZD1222: *n* = 2), 17 (BNT162b2: *n* = 15, AZD1222: *n* = 2) and 43 (BNT162b2: *n* = 43) after the first, second and third vaccines, respectively.

	After the First Vaccine Dose	Number of Cases	After the Second Vaccine Dose	Number of Cases	After the Third Vaccine Dose	Number of Cases
*Local reactions:*	Pain	7	Pain	7	Pain	9
	Erythema	0	Erythema	1	Erythema	2
	Swelling	0	Swelling	1	Swelling	4
	Pruritus	0	Pruritus	1	Pruritus	0
	Tingling	0	Tingling	0	Tingling	0
*Systemic reactions:*	Fever	1	Fever	1	Fever	1
	Nausea	0	Nausea	1	Nausea	1
	Vomiting	0	Vomiting	0	Vomiting	0
	Rhinorrhea	0	Rhinorrhea	0	Rhinorrhea	1
	Cough	0	Cough	0	Cough	0
	Myalgia	2	Myalgia	3	Myalgia	7
	Arthralgia	0	Arthralgia	0	Arthralgia	5
	Chills	1	Chills	1	Chills	3
	Malaise	0	Malaise	0	Malaise	3
	Headache	2	Headache	1	Headache	3
	Allergic reaction	0	Allergic reaction	0	Allergic reaction	0
	Dizziness	0	Dizziness	0	Dizziness	1
	Throat pain	0	Throat pain	0	Throat pain	2
	Chest pain or palpitations	0	Chest pain or palpitations	0	Chest pain or palpitations	0
	Diarrhea	0	Diarrhea	0	Diarrhea	1
	Pruritus	0	Pruritus	0	Pruritus	0
	Lack of appetite	0	Lack of appetite	0	Lack of appetite	0
	Local lymphadenopathy	0	Local lymphadenopathy	0	Local lymphadenopathy	0
	High blood pressure	1	High blood pressure	0	High blood pressure	0
	Uveitis	0	Uveitis	0	Uveitis	0
	Herpes zoster	0	Herpes zoster	0	Herpes zoster	0
	Pericarditis	0	Pericarditis	0	Pericarditis	0
	Vaginal bleeding	0	Vaginal bleeding	0	Vaginal bleeding	0

**Table 2:** Demographic and clinical characteristics of the homologous, heterologous booster, and infection as a subgroup of SARS-CoV-2 vaccination of patients with aiRMDs.

	Age, (Years)	Female	Disease Duration, (Years)	Therapy			
				GC	cDMARD	bDMARD	JAKi
All patients with aiRMDs, *n* = 46	65 ± 10	33 (72%)	9.7 ± 7.2	6	24	20	1
Homologous booster vaccination group, *n* = 22	63 ± 10	17 (77%)	8.5 ± 6.2	1	14	12	1
RA, *n* = 12	65 ± 10	12 (100%)	8.4 ± 7.2	0	6	8	1
PsA, *n* = 3	56 ± 14	1 (33%)	8.7 ± 3.5	1	2	2	0
AxSpa, *n* = 1	68	0 (0%)	8	0	0	1	0
SLE, *n* = 1	66	1 (100%)	4	0	1	0	0
SS, *n* = 3	57 ± 9	3 (100%)	10 ± 8.7	0	3	1	0
LVV, *n* = 1	73	1 (100%)	5	0	1	0	0
AAV, *n* = 1	69	0 (0%)	9	0	1	0	0
Heterologous booster vaccination group, *n* = 12	59 ± 10	7 (58%)	11.2 ± 7.7	1	10	5	0
RA, *n* = 8	60 ± 5	8 (100%)	10.9 ± 6.6	1	6	5	0
PsA, *n* = 2	56 ± 29	1 (50%)	3.5 ± 0.7	0	2	0	0
SLE, *n* = 1	54	1 (100%)	26	0	1	0	0
Other vasculitis, *n* = 1	55	1 (100%)	14	0	1	0	0
Infection group, *n* = 12	59 ± 9	9 (75%)	10.5 ± 8.6	4	8	6	0
RA, *n* = 5	56 ± 11	4 (80%)	13 ± 10.6	0	4	3	0
PsA, *n* = 2	55 ± 1	1 (50%)	5 ± 1.4	0	2	2	0
SLE, *n* = 1	69	1 (100%)	22	1	1	0	0
SS, *n* = 1	59	1 (100%)	14	1	1	0	0
LVV, *n* = 2	68 ± 12	1 (50%)	5 ± 5.7	1	2	0	0
AAV, *n* = 1	55	1 (100%)	5	1	0	1	0

## Data Availability

Research Data are available upon request from the corresponding author.

## References

[1] Shalash A.O., Toth I., Skwarczynski M. (2022). The potential of developing a protective peptide-based vaccines against SARS-CoV-2. Drug Dev. Res..

[2] Jena A., Mishra S., Deepak P., Kumar M.P., Sharma A., Patel Y.I., Kennedy N.A., Kim A.H.J., Sharma V., Sebastian S. (2022). Response to SARS-CoV-2 vaccination in immune mediated inflammatory diseases: Systematic review and meta-analysis. Autoimmun Rev..

[3] Baden L.R., El Sahly H.M., Essink B., Kotloff K., Frey S., Novak R., Diemert D., Spector S.A., Rouphael N., Creech C.B. (2021). Efficacy and Safety of the mRNA-1273 SARS-CoV-2 Vaccine. N. Engl. J. Med..

[4] Ramasamy M.N., Minassian A.M., Ewer K.J., Flaxman A.L., Folegatti P.M., Owens D.R., Voysey M., Aley P.K., Angus B., Babbage G. (2021). Safety and immunogenicity of ChAdOx1 nCoV-19 vaccine administered in a prime-boost regimen in young and old adults (COV002): A single-blind, randomised, controlled, phase 2/3 trial. Lancet.

[5] Benucci M., Damiani A., Gobbi F.L., Lari B., Grossi V., Infantino M., Manfredi M. (2022). Role of booster with BNT162b2 mRNA in SARS-CoV-2 vaccination in patients with rheumatoid arthritis. Immunol. Res..

[6] Belleudi V., Rosa A.C., Poggi F.R., Armuzzi A., Nicastri E., Goletti D., Diamanti A.P., Davoli M., Agabiti N., Addis A. (2021). Direct and Indirect Impact of COVID-19 for Patients with Immune-Mediated Inflammatory Diseases: A Retrospective Cohort Study. J. Clin. Med..

[7] Landewe R.B., Machado P.M., Kroon F., Bijlsma H.W., Burmester G.R., Carmona L., Combe B., Galli M., Gossec L., Iagnocco A. (2020). EULAR provisional recommendations for the management of rheumatic and musculoskeletal diseases in the context of SARS-CoV-2. Ann. Rheum. Dis..

[8] Fragoulis G.E., Karamanakos A., Arida A., Tektonidou M.G. (2022). Clinical outcomes of breakthrough COVID-19 after booster vaccination in patients with systemic rheumatic diseases. Rmd Open.

[9] Fragoulis G.E., Bournia V.K., Mavrea E., Evangelatos G., Fragiadaki K., Karamanakos A., Kravariti E., Laskari K., Panopoulos S., Pappa M. (2022). COVID-19 vaccine safety and nocebo-prone associated hesitancy in patients with systemic rheumatic diseases: A cross-sectional study. Rheumatol. Int..

[10] Connolly C.M., Ruddy J.A., Boyarsky B.J., Barbur I., Werbel W.A., Geetha D., Garonzik-Wang J.M., Segev D.L., Christopher-Stine L., Paik J.J. (2022). Disease Flare and Reactogenicity in Patients With Rheumatic and Musculoskeletal Diseases Following Two-Dose SARS-CoV-2 Messenger RNA Vaccination. Arthritis Rheumatol.

[11] Papagoras C., Fragoulis G.E., Zioga N., Simopoulou T., Deftereou K., Kalavri E., Zampeli E., Gerolymatou N., Kataxaki E., Melissaropoulos K. (2022). Better outcomes of COVID-19 in vaccinated compared to unvaccinated patients with systemic rheumatic diseases. Ann. Rheum. Dis..

[12] Szebeni G.J., Gemes N., Honfi D., Szabo E., Neuperger P., Balog J.A., Nagy L.I., Szekanecz Z., Puskas L.G., Toldi G. (2022). Humoral and Cellular Immunogenicity and Safety of Five Different SARS-CoV-2 Vaccines in Patients With Autoimmune Rheumatic and Musculoskeletal Diseases in Remission or With Low Disease Activity and in Healthy Controls: A Single Center Study. Front. Immunol..

[13] Szekanecz Z., Balog A., Constantin T., Czirjak L., Geher P., Kovacs L., Kumanovics G., Nagy G., Rakoczi E., Szamosi S. (2022). COVID-19: Autoimmunity, multisystemic inflammation and autoimmune rheumatic patients. Expert Rev. Mol. Med..

[14] Vokó Z., Kiss Z., Surján G., Surján O., Barcza Z., Wittmann I., Molnár G.A., Nagy D., Müller V., Bogos K. (2022). Effectiveness and Waning of Protection with Different SARS-CoV-2 Primary and Booster Vaccines Dur-ing the Delta Pandemic Wave in 2021 in Hungary (HUN-VE 3 Study). Front. Immunol..

[15] Goldberg Y., Mandel M., Bar-On Y.M., Bodenheimer O., Freedman L., Haas E.J., Milo R., Alroy-Preis S., Ash N., Huppert A. (2021). Waning Immunity after the BNT162b2 Vaccine in Israel. N. Engl. J. Med..

[16] Barda N., Dagan N., Cohen C., Hernan M.A., Lipsitch M., Kohane I.S., Reis B.Y., Balicer R.D. (2021). Effectiveness of a third dose of the BNT162b2 mRNA COVID-19 vaccine for preventing severe outcomes in Israel: An observational study. Lancet.

[17] Munro A.P.S., Janani L., Cornelius V., Aley P.K., Babbage G., Baxter D., Bula M., Cathie K., Chatterjee K., Dodd K. (2021). Safety and immunogenicity of seven COVID-19 vaccines as a third dose (booster) following two doses of ChAdOx1 nCov-19 or BNT162b2 in the UK (COV-BOOST): A blinded, multicentre, randomised, controlled, phase 2 trial. Lancet.

[18] Chenchula S., Karunakaran P., Sharma S., Chavan M. (2022). Current evidence on efficacy of COVID-19 booster dose vaccination against the Omicron variant: A systematic review. J. Med. Virol..

[19] Bieber A., Sagy I., Novack L., Brikman S., Abuhasira R., Ayalon S., Novofastovski I., Abu-Shakra M., Mader R. (2022). BNT162b2 mRNA COVID-19 vaccine and booster in patients with autoimmune rheumatic diseases: A national cohort study. Ann. Rheum. Dis..

[20] Beyer D.K., Forero A. (2022). Mechanisms of Antiviral Immune Evasion of SARS-CoV-2. J. Mol. Biol..

[21] Gu W., Gan H., Ma Y., Xu L., Cheng Z.J., Li B., Zhang X., Jiang W., Sun J., Sun B. (2022). The molecular mechanism of SARS-CoV-2 evading host antiviral innate immunity. Virol. J..

[22] Pusnik J., Richter E., Schulte B., Dolscheid-Pommerich R., Bode C., Putensen C., Hartmann G., Alter G., Streeck H. (2021). Memory B cells targeting SARS-CoV-2 spike protein and their dependence on CD4(+) T cell help. Cell Rep..

[23] Dulic S., Vasarhelyi Z., Sava F., Berta L., Szalay B., Toldi G., Kovacs L., Balog A. (2017). T-Cell Subsets in Rheumatoid Arthritis Patients on Long-Term Anti-TNF or IL-6 Receptor Blocker Therapy. Mediat. Inflamm..

[24] Dulic S., Vasarhelyi Z., Bajnok A., Szalay B., Toldi G., Kovacs L., Balog A. (2018). The Impact of Anti-TNF Therapy on CD4+ and CD8+ Cell Subsets in Ankylosing Spondylitis. Pathobiology.

[25] Dulic S., Toldi G., Sava F., Kovacs L., Molnar T., Milassin A., Farkas K., Rutka M., Balog A. (2020). Specific T-Cell Subsets Can Predict the Efficacy of Anti-TNF Treatment in Inflammatory Bowel Diseases. Arch. Immunol. Exp..

[26] Matula Z., Gonczi M., Beko G., Kadar B., Ajzner E., Uher F., Valyi-Nagy I. (2022). Antibody and T Cell Responses against SARS-CoV-2 Elicited by the Third Dose of BBIBP-CorV (Sinopharm) and BNT162b2 (Pfizer-BioNTech) Vaccines Using a Homologous or Heterologous Booster Vaccination Strategy. Vaccine.

[27] Meng H., Mao J., Ye Q. (2022). Booster vaccination strategy: Necessity, immunization objectives, immunization strategy, and safety. J. Med. Virol..

[28] Arbel R., Hammerman A., Sergienko R., Friger M., Peretz A., Netzer D., Yaron S. (2021). BNT162b2 Vaccine Booster and Mortality Due to COVID-19. N. Engl. J. Med..

[29] Manisty C., Otter A.D., Treibel T.A., McKnight A., Altmann D.M., Brooks T., Noursadeghi M., Boyton R.J., Semper A., Moon J.C. (2021). Antibody response to first BNT162b2 dose in previously SARS-CoV-2-infected individuals. Lancet.

[30] Aletaha D., Neogi T., Silman A.J., Funovits J., Felson D.T., Bingham C.O., Birnbaum N.S., Burmester G.R., Bykerk V.P., Cohen M.D. (2010). 2010 Rheumatoid arthritis classification criteria: An American College of Rheumatology/European League Against Rheumatism collaborative initiative. Arthritis Rheum..

[31] Taylor W., Gladman D., Helliwell P., Marchesoni A., Mease P., Mielants H., Group C.S. (2006). Classification criteria for psoriatic arthritis: Development of new criteria from a large international study. Arthritis Rheum..

[32] Rudwaleit M., van der Heijde D., Landewe R., Listing J., Akkoc N., Brandt J., Braun J., Chou C.T., Collantes-Estevez E., Dougados M. (2009). The development of Assessment of SpondyloArthritis international Society classification criteria for axial spondyloarthritis (part II): Validation and final selection. Ann. Rheum. Dis..

[33] Hochberg M.C. (1997). Updating the American College of Rheumatology revised criteria for the classification of systemic lupus erythematosus. Arthritis Rheum..

[34] Petri M., Orbai A.M., Alarcon G.S., Gordon C., Merrill J.T., Fortin P.R., Bruce I.N., Isenberg D., Wallace D.J., Nived O. (2012). Derivation and validation of the Systemic Lupus International Collaborating Clinics classification criteria for systemic lupus erythematosus. Arthritis Rheum..

[35] Jennette J.C., Falk R.J., Bacon P.A., Basu N., Cid M.C., Ferrario F., Flores-Suarez L.F., Gross W.L., Guillevin L., Hagen E.C. (2013). 2012 revised International Chapel Hill Consensus Conference Nomenclature of Vasculitides. Arthritis Rheum..

[36] Shiboski C.H., Shiboski S.C., Seror R., Criswell L.A., Labetoulle M., Lietman T.M., Rasmussen A., Scofield H., Vitali C., Bowman S.J. (2017). 2016 American College of Rheumatology/European League Against Rheumatism Classification Criteria for Primary Sjogren’s Syndrome: A Consensus and Data-Driven Methodology Involving Three International Patient Cohorts. Arthritis Rheumatol..

[37] International Team for the Revision of the International Criteria for Behçet’s Disease (2014). The International Criteria for Behçet’s Disease (ICBD): A collaborative study of 27 countries on the sensitivity and specificity of the new criteria. J. Eur. Acad Derm. Venereol.

[38] Irsara C., Egger A.E., Prokop W., Nairz M., Loacker L., Sahanic S., Pizzini A., Sonnweber T., Holzer B., Mayer W. (2021). Clinical validation of the Siemens quantitative SARS-CoV-2 spike IgG assay (sCOVG) reveals improved sensitivity and a good correlation with virus neutralization titers. Clin. Chem. Lab. Med..

[39] Saiag E., Goldshmidt H., Sprecher E., Ben-Ami R., Bomze D. (2021). Immunogenicity of a BNT162b2 vaccine booster in health-care workers. Lancet Microbe.

